# Exploring the theranostic potential of two metabolically stable GRPR-targeting peptides labelled with Ga-68 for PET imaging

**DOI:** 10.1186/s41181-026-00431-5

**Published:** 2026-02-17

**Authors:** Karim Obeid, Ekaterina Bezverkhniaia, Vladimir Tolmachev, Anna Orlova, Panagiotis Kanellopoulos

**Affiliations:** 1 Department of Medicinal Chemistry, Uppsala University, 751 83 Uppsala, Sweden; 2 Department of Immunology, Genetics and Pathology, Uppsala University, 751 83 Uppsala, Sweden; 3 Science for Life Laboratory, Uppsala University, 752 37 Uppsala, Sweden

**Keywords:** GRPR, Radiopharmaceuticals, ^68^Ga, PET, Prostate cancer, Bombesin, Imaging

## Abstract

**Background:**

Gastrin-releasing peptide receptor (GRPR) attracts increasing attention as a target for radiotheranostic applications. We previously developed a metabolically stable GRPR-targeting peptide, incorporating α-methyl-L-tryptophan within its sequence (PEG_2_-Pip-D-Phe^6^-Gln^7^-MetTrp^8^-Ala^9^-Val^10^-Sar^11^-His^12^-Sta^13^-Leu^14^-NH_2_) and coupled it to DOTAGA chelator (PKB2) and to DOTA (PKB3). When labelled with Lu-177, both peptide variants demonstrated promising properties for targeted radionuclide therapy.

**Results:**

In this study, we aimed to evaluate the diagnostic counterparts of PKB2 and PKB3 by radiolabelling them with Ga-68 for positron emission tomography (PET) imaging. [^68^Ga]Ga-PKB2 and [^68^Ga]Ga-PKB3 were produced with radiochemical yields over 99% and radiochemical purities over 97%. Both radiopeptides showed a high GRPR affinity with IC_50_ values in the low nanomolar range and a GRPR-mediated uptake in PC-3 cells with slow internalization. The labelled peptides [^68^Ga]Ga-PKB2 and [^68^Ga]Ga-PKB3 demonstrated fast clearance with activity concentration in blood below 0.5%IA/g at 2 pi, and a high tumour activity uptake in PC-3 xenografts (16 ± 3%IA/g and 17 ± 2%IA/g, respectively). [^68^Ga]Ga-PKB3 had a significantly higher activity uptake in the pancreas (GRPR-expressing organ) and lower uptake in the kidneys than [^68^Ga]Ga-PKB2. PET/CT images were concordant with the biodistribution results, clearly delineating tumour tissue.

**Conclusions:**

[^68^Ga]Ga-PKB2 and [^68^Ga]Ga-PKB3 are promising PET tracers for imaging of GRPR-positive tumours and are potential diagnostic counterparts to their ^177^Lu-labelled analogues, supporting their use as a ^177^Lu/^68^Ga theranostic pair.

**Supplementary Information:**

The online version contains supplementary material available at 10.1186/s41181-026-00431-5.

## Introduction

Radiotheranostics is an emerging field in oncology that combines diagnostic imaging with targeted therapy. This allows clinicians to visualize tumour-specific targets (e.g. overexpressed receptors) and to select patients who are most likely to benefit from targeted radionuclide therapy (TRT) (Aboagye et al. 2023; Bodei et al. 2022; Sgouros et al. 2020). Target-specific radiopeptides suitable for single-photon emission computed tomography (SPECT) or positron emission tomography (PET) are used to visualise the level of the target expression in a tumour. The same or a similar molecule can be labelled with a therapeutic radionuclide to selectively eradicate cancer cells if the molecular target is present.

The gastrin-releasing peptide receptor (GRPR) is an attractive target for radiotheranostic applications. It is overexpressed in many human malignancies, including prostate, breast, lung, colon, and neuroendocrine cancers (Reubi et al. 2002; Patel et al. 2006; Ma and Gao 2024; Carroll et al. 1999). The development of GRPR-targeting peptides was initially based on the receptor agonist bombesin (BBN). GRPR agonists were associated with severe side effects in clinical settings (Bodei et al. 2007). This led to a shift towards BBN-based antagonists, which demonstrated superior targeting properties and reduced risk of side effects compared to agonists (Mansi et al. 2021; Cescato et al. 2008). Despite these advances, GRPR-targeting peptides suffer from rapid degradation by proteolytic enzymes, such as the neutral endopeptidase or neprilysin (NEP), which reduces their efficacy and impairs clinical translation. Therefore, there remains a need to develop GRPR antagonists with high metabolic stability, as resistance to enzymatic degradation is crucial for achieving high tumour activity uptake and favourable pharmacokinetics (Shipp et al. 1991; Lymperis et al. 2018; Zhang et al. 2004). Several studies have demonstrated that the administration of NEP inhibitors in rodents results in improved metabolic stability and increased tumour uptake of GRPR-targeting radiopharmaceuticals (Marsouvanidis et al. 2014; Chatalic et al. 2016). However, administering multiple agents complicates approval of imaging or therapeutic protocols in clinical settings. Alternatively, chemical modifications in peptide sequences can protect the ligands from enzymatic degradation (Evans et al. 2020). Such modifications typically include incorporation of methylated or unnatural amino acids in the major cleavage sites within the GRPR-targeting peptides (e.g. Gly^11^-His^12^ and Gln^7^-Trp^8^ bonds) (Teufel et al. 2003; Linder et al. 2009). These changes can be problematic, as they may interfere with ligand-receptor interactions, posing challenges in molecular optimization. For instance, the exchange of Gly^11^ with DAla^11^ improved the metabolic stability of [^111^In]In-SB4, but resulted in reduced GRPR affinity compared to its parent compound [^111^In]In-SB3 (Lymperis et al. 2019). Previous studies showed that the incorporation of sarcosine (Sar, N-methyl-glycine) in position 11 of the GRPR targeting sequences of [^99m^Tc]Tc-DB15 and [^111^In]In-AU-SAR-M1 resulted in an enhanced metabolic stability while maintaining high receptor affinity (Nock et al. 2021; Abouzayed et al. 2023). A similar effect has been observed with the incorporation of α-methyl-L-tryptophan (MetTrp) at position 8 of [^177^Lu]Lu-AMTG peptide sequence (Günther et al. 2022).

**Fig. 1:**
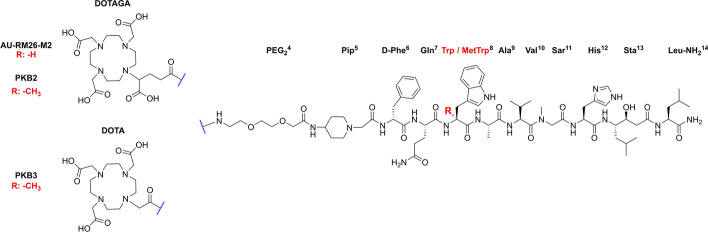
Chemical structures of AU-RM26-M2 [DOTAGA-PEG_2_-Pip-[Sar^11^]RM26], PKB2 [DOTAGA-PEG_2_-Pip-[MetTrp^8^, Sar^11^]RM26], and PKB3 [DOTA-PEG_2_-Pip-[MetTrp^8^, Sar^11^]RM26]. DOTAGA: (1,4,7,10-tetra(carboxymethyl)-1,4,7,10-tetraazacyclo-dodecane-1-glutaric acid); DOTA: 1,4,7,10-tetraazacyclododecane-1,4,7,10-tetraacetic acid]; Pip: 4-amino-1-carboxymethyl-piperidine; PEG_2_: 8-amino-3,6 dioxa-octanoic acid; RM26: D-Phe^6^-Gln^7^-Trp^8^-Ala^9^-Val^10^-Gly^11^-His^12^-Sta^13^-Leu^14^-NH_2_

We have recently developed two metabolically stable GRPR-targeting peptides, incorporating both the MetTrp^8^ and Sar^11^ modifications in their peptide sequence: PKB2, carrying a DOTAGA chelator, and PKB3, its DOTA counterpart (Fig. 1). Both of these chelators are suitable for stable chelation of trivalent metals, such as Ga-68 for PET, In-111 for SPECT imaging, or Y-90, Lu-177, and Ac-225 for TRT (Wadas et al. 2010; Parus et al. 2015; Thiele and Wilson 2018).

The two variants were previously labelled with the therapeutic radionuclide Lu-177 and demonstrated a promising biodistribution profile for TRT; i.e., low activity uptake in the kidneys and high tumour activity uptake already 2 h post-injection (pi) (16 ± 4%IA/g and 24 ± 3%IA/g, for [^177^Lu]Lu-PKB2 and [^177^Lu]Lu-PKB3), and high retention in tumours up to 24 h pi (Obeid et al. 2025). The metabolic stability of [^177^Lu]Lu-PKB2 and [^177^Lu]Lu-PKB3 was also improved, with 85% and 91% intact peptide detected in blood circulation 5 min pi, respectively.

As mentioned, both PKB2 and PKB3 carry chelators suitable for radiolabelling with the positron-emitting radionuclide Ga-68, and thus they could be used for the visualisation of GRPR expression in lesions before TRT. Therefore, this study aimed to evaluate [^68^Ga]Ga-PKB2 and [^68^Ga]Ga-PKB3 as components of a potential ^177^Lu/^68^Ga theranostic pair. [^68^Ga]Ga-AU-RM26-M2 (DOTAGA-PEG_2_-Pip-[Sar^11^]RM26) (Obeid et al. 2024; Kanellopoulos et al. 2025), which contains the Gly^11^/Sar^11^ but not the Trp^8^/MetTrp^8^ substitution, was used as a comparator only in vitro on GRPR-positive PC-3 cells. In vivo performance of [^68^Ga]Ga-PKB2 and [^68^Ga]Ga-PKB3 was compared in PC-3 xenograft-bearing mice.

## Materials and methods

### Peptides and reagents

The peptides AU-RM26-M2, PKB2, PKB3, and NOTA-PEG2-RM26 were synthesized by Pepmic Co., Ltd. (Suzhou, China). [^125^I]I-Tyr^4^-BBN was supplied by Revvity (Boston, MA, USA). The human GRPR-positive prostate cancer cell line (PC-3) was purchased from American Type Culture Collection (Manassas, VA, USA). The cell culture medium used was Roswell Park Memorial Institute (RPMI) 1640 (containing L-glutamine), supplemented with 10% fetal bovine serum and 1% penicillin–streptomycin (100 IU/mL penicillin, 100 µg/mL streptomycin). Trypsin–EDTA (0.25%) was used for cell detachment. Media supplements and the trypsin solution were purchased from Biochrom AG (Berlin, Germany). Ga-68 was eluted with metal-free 0.1 M HCl through a ^68^Ge/^68^Ga generator (Eckert & Ziegler, Berlin, Germany). All other reagents used were of chemical grade.

### Labelling and quality control

The labelling of AU-RM26-M2, PKB2, and PKB3 with Ga-68 was performed by adding 250 µL of freshly eluted Ga-68 (70–80 MBq, measurement after labelling) to a solution containing 5 nmol of peptide, 125 µL EtOH (99.5%), and 30 µL NH_4_OAc (1 M, pH 4.5). The reaction mixture was incubated at 80 °C for 20 min.

The radiochemical yields of the labelled analogues were assessed using instant thin-layer chromatography (iTLC) with 0.2 M citric acid as the mobile phase and silica gel chromatography paper (Agilent Technologies, Santa Clara, CA, USA) as the stationary phase. The iTLC results were analysed with the Cyclone® Plus Phosphorimager (Revvity, Sweden) (n = 4 for [^68^Ga]Ga-PKB2 and [^68^Ga]Ga-PKB3 and n = 2 for [^68^Ga]Ga-AU-RM26-M2).

The radiochemical purity (RCP) of the radiopeptides was determined using reverse-phase high-performance liquid chromatography (HPLC). The system included a LaPrep Sigma HPLC LP1100 pump (Hitachi High-Tech Corporation, Hitachinaka, Ibaraki, Japan), a 40D LWL UV detector with a 4 µL flow cell (Knauer, Berlin, Germany), a flow-scan radioactivity detector (Bioscan) with an FC-3300 NaI/PMT radioactivity probe (Eckert & Ziegler, Berlin, Germany), and a manual Rheodyne 7725i injector fitted with a 20 µL loop (IDEX Health & Science, LLC, Rohnert Park, CA, USA). The column used was a Luna® Omega C18 column (5 μm, 100 Å, 100 × 4.6 mm, Phenomenex, Værløse, Denmark). The gradient elution started at 95% 0.1% v/v aqueous trifluoroacetic acid (TFA)/5% 0.1% v/v TFA in acetonitrile (MeCN) and reached 40% TFA/60% MeCN over 20 min.

Radiometal-chelate stability was determined using iTLC (as described above) after the radiopeptides (C_f_ = 1 nM) were challenged with a 1000-fold molar excess of ethylenediaminetetraacetic acid (Na_2_EDTA) at 37.4 °C for 1 h (n = 4).

The loading with ^nat^Ga was performed by adding 30 µL GaCl_3_ (15 mM) to 30 nmol peptide, 150 µL HCl (0.1 M), 125 µL EtOH, and 30 µL of NH_4_OAc (1 M, pH 4.5). The reaction was carried out at 70 °C for 60 min.

### In vitro assays

#### In vitro specificity and cellular uptake

In vitro specificity and cellular uptake assays for [^68^Ga]Ga-AU-RM26-M2, [^68^Ga]Ga-PKB2, and [^68^Ga]Ga-PKB3 were performed in parallel and in triplicate for each compound.

To determine specificity to GRPR, PC-3 cells were seeded into 6-well plates (8 × 10^5^ cells/well). The following day, the media were removed, and the cells were washed with phosphate-buffered saline containing 1% w/v bovine serum albumin (PBS/BSA). The radioligand under investigation was added to all wells (C_f_ = 1 nM), while “blocked” wells were also treated with the GRPR-blocking agent NOTA-PEG_2_-RM26 (C_f_ = 100 nM). After incubating at 37 °C for 1 h, the supernatant was removed, and the cells were washed and collected by treating them twice with 500 µL NaOH (1 M). The radioactivity content was measured using the Wizard2™ gamma counter (PerkinElmer, Waltham, MA, USA). Statistical analysis was determined by applying two-way ANOVA with Tukey’s post hoc analysis using GraphPad Prism v10.

For the investigation of cellular uptake and internalisation, 8 × 10^5^ PC-3 cells were seeded into 35 mm Petri dishes. The following day, after washing with PBS/BSA, the cells were incubated at 37 °C with 1 mL of the test radioligand solution (1 nM peptide in PBS/BSA). At 1, 2, and 3-h time points, the cells were washed and treated twice with an acid wash (0.2 M glycine buffer with 0.15 M NaCl and 4 M urea, pH 2), incubated on ice for 5 min, and the supernatant was collected (membrane-bound activity). The cells were then washed and collected by treating them twice with 1 M NaOH (internalized activity). The activity content was measured with the gamma counter.

#### Competitive binding assay

For the competitive binding assay, 1 × 10^5^ PC-3 cells were seeded into 12-well plates and allowed to proliferate overnight. The next day, the media was discarded, and the cells were washed with cold PBS/BSA. Subsequently, 350 μL cold PBS/BSA, 100 μL [^125^I]I-Tyr^4^-BBN (24.6 fmol), and 50 μL of the test ligand (at concentrations ranging from 0.01 nM to 100 nM) were added to each well. The cells were incubated at 4 °C for 5 h to allow the binding equilibrium to be reached. After incubation, the cells were washed with cold PBS/BSA and collected by treating them twice with 500 μL of 1 M NaOH. The radioactivity was quantified using the gamma counter, and the resulting data were analysed using a nonlinear regression model analysis in GraphPad Prism v10. This assay was performed for ^nat^Ga-AU-RM26-M2, as well as for ^nat^Ga-PKB2 and ^nat^Ga-PKB3 (n = 6).

### In vivo studies

All animal studies were conducted following European guidelines for the protection of laboratory animals and were approved by the regional ethical committee on animal experiments in Uppsala, Sweden (permit no. 5.8.18–00473/21). Biodistribution studies and PET/CT imaging were performed using BALB/c nu/nu mice xenografted with 7 × 10^6^ PC-3 cells on the right hind leg. In vivo experiments were conducted 4 weeks after inoculation when the average animal weight was 17 ± 1.5 g, and the average tumour weight was 0.3 ± 0.1 g.

#### Biodistribution and PET/CT images

Biodistribution studies of [^68^Ga]Ga-PKB2 and [^68^Ga]Ga-PKB3 were performed 2 h post-injection (pi). Xenografted mice (n = 4 per radiopeptide) were intravenously injected via the tail vein with 100 µL of a solution containing the test radioligand in PBS/BSA (500 kBq, 100 pmol peptide). At the designated time point, the mice were euthanized, organs of interest were collected and weighed, and their radioactivity content was measured using the gamma counter. The percentage of injected activity per gram (%IA/g) was calculated for the collected organs and tumours, and the percentage of injected activity (%IA) was determined for the gastrointestinal tract (excluding stomach, pancreas and part of the small intestine which were excised and measured separately) and the rest of the carcass. Statistical analysis was performed using GraphPad Prism 7 with a two-way ANOVA (Šidák post hoc analysis).

PET/CT imaging of [^68^Ga]Ga-PKB2 and [^68^Ga]Ga-PKB3 was performed on two separate PC-3 xenograft-bearing mice. The animals were injected with 100 µL of a solution containing the test radioligand in PBS/BSA (100 pmol peptide corresponding to 1.3 MBq for [^68^Ga]Ga-PKB2 and 1.5 MBq for [^68^Ga]Ga-PKB3). At 2 h pi, PET/CT images were acquired using the Nano PET 3 T (PET/MRI) and nanoScan (SPECT/CT) systems (Mediso Medical Imaging Systems, Budapest, Hungary). Nucline nanoScan 3.04.014.0000 software was employed for the reconstruction of the PET scans, while the Filter Back Projection method in Nucline 2.03 software was used for the CT scan reconstructions. The PET and CT images were fused using Nucline 2.03 software and are shown on the RGB colour scale as maximum intensity projections.

Molar activities were adjusted when needed using the corresponding unlabelled compound.

## Results

### Labelling and quality control

**Fig. 2:**
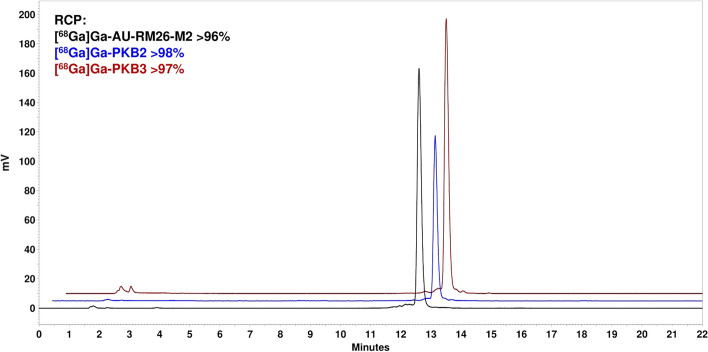
Radiochromatograms of [^68^Ga]Ga-AU-RM26-M2 (black), [^68^Ga]Ga-PKB2 (blue), and [^68^Ga]Ga-PKB3 (red)

The radiochemical yields of [^68^Ga]Ga-AU-RM26-M2, [^68^Ga]Ga-PKB2 and [^68^Ga]Ga-PKB3, determined by iTLC, were > 99% (n = 4 for [^68^Ga]Ga-PKB2 and [^68^Ga]Ga-PKB3 and n = 2 for [^68^Ga]Ga-AU-RM26-M2) with molar activities in the range of 12–15 MBq/nmol. The release of Ga-68 for all radiopeptides was negligible after the challenge with a 1000-fold molar excess of EDTA, with 98 ± 1% (n = 4) of the activity remaining bound to the corresponding peptide. The radiochemical purity, determined by radio-HPLC, was > 96% (Fig. 2).

### In vitro assays

#### In vitro specificity and cellular uptake

**Fig. 3:**
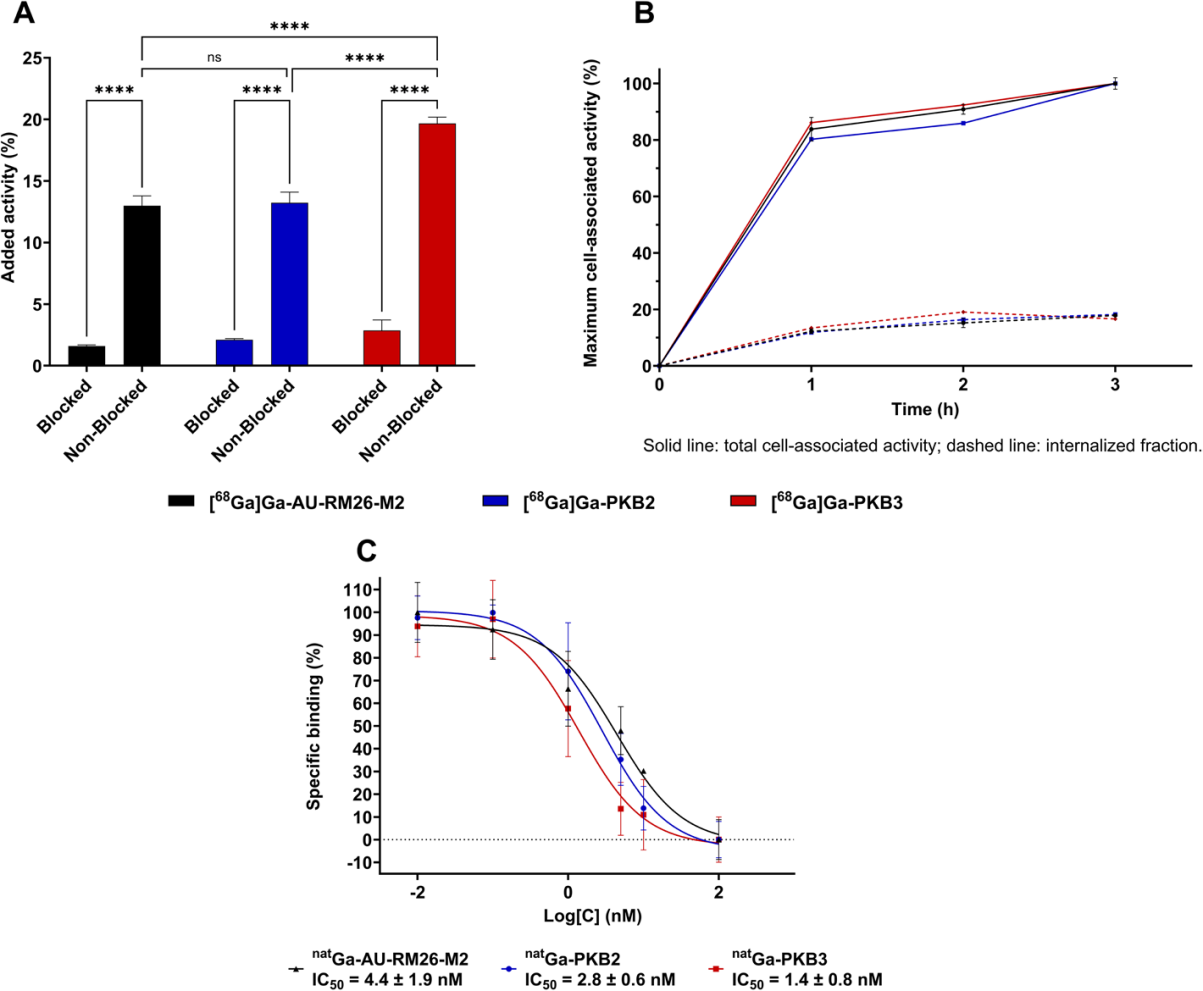
In vitro binding specificity (**A**) and cellular internalization (**B**) of [^68^Ga]Ga-AU-RM26-M2 (black), [^68^Ga]Ga-PKB2 (blue), and [^68^Ga]Ga-PKB3 (red) in PC-3 cells. Representative competition binding curves for ^nat^Ga-AU-RM26-M2, ^nat^Ga-PKB2, and ^nat^Ga-PKB3 against [^125^I]I-Tyr^4^-BBN in PC-3 cells (**C**). Error bars could not be visible due to low variability. Data are presented as mean ± SD, n = 3 for **A** and **B**; n = 6 for **C**

The in vitro specificity test for [^68^Ga]Ga-AU-RM26-M2, [^68^Ga]Ga-PKB2, and [^68^Ga]Ga-PKB3 showed a significantly (*p* < 0.0001 in all cases) higher activity bound to cells with non-blocked receptors compared to the cells pre-blocked with an excess of non-labelled GRPR ligand (Fig. 3A). This demonstrated a specific binding to GRPR for all three compounds, with [^68^Ga]Ga-PKB3 having significantly (*p* < 0.0001) higher cellular uptake than [^68^Ga]Ga-AU-RM26-M2 and [^68^Ga]Ga-PKB2. The experiment for the three radiopeptides was done in parallel on the same batch of cells.

All three radiopeptides demonstrated a very similar pattern of cellular uptake with rapid binding and a slow internalization (Fig. 3B). Within 3 h of continuous incubation, 17–18% of the cell-associated activity was internalized.

#### Competitive binding assay

The three peptides loaded with natural gallium (^nat^Ga) displayed an IC_50_ in the single-digit nanomolar range. ^nat^Ga-PKB3 showed the strongest binding affinity, followed by ^nat^Ga-PKB2, and ^nat^Ga-AU-RM26-M2 (Fig. 3C).

### In vivo studies

#### Biodistribution and PET/CT images

**Fig. 4:**
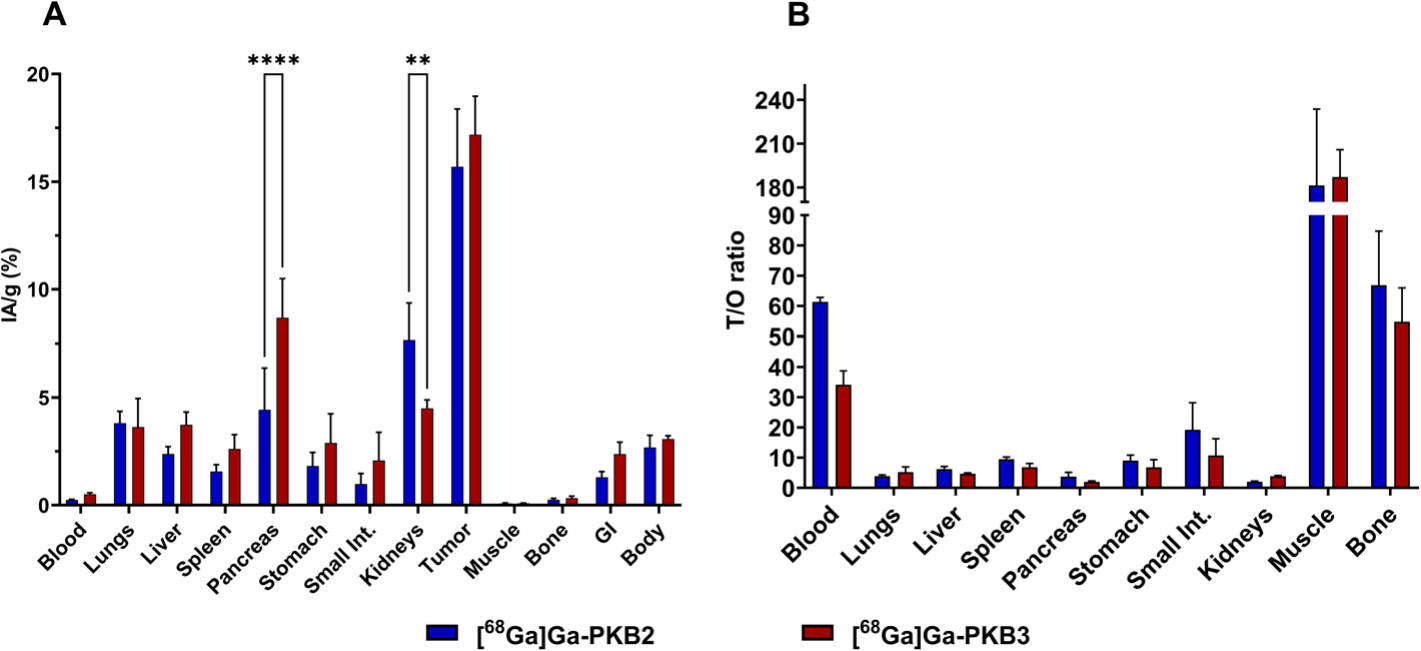
Biodistributions (**A**) and tumour-to-organ ratios (**B**) of [^68^Ga]Ga-PKB2 (blue) and [^68^Ga]Ga-PKB3 (red) at 2 h pi in PC-3 xenografted mice. Data are presented as mean ± SD (n = 4). ** indicates *p* < 0.01, and **** indicates *p* < 0.0001. The gastrointestinal tract (without stomach, pancreas and part of the small intestine) and the rest of the carcass values are given as %IA

The biodistribution profile and tumour-to-organ ratios (T/O) of [^68^Ga]Ga-PKB2 and [^68^Ga]Ga-PKB3 at 2 h pi are shown in Figs. 4A and B, respectively. [^68^Ga]Ga-PKB2 and [^68^Ga]Ga-PKB3 demonstrated a fast blood clearance with less than 0.5%IA/g at the studied time point, low background uptake in non-target organs, including the lungs, liver, spleen, stomach, and small intestine (< 4%IA/g), and negligible uptake in muscles and bones (< 0.3%IA/g). The excretion was mainly renal, with a moderate activity uptake in the kidneys. The activity uptake in kidneys for [^68^Ga]Ga-PKB2 was 1.8-fold higher compared to [^68^Ga]Ga-PKB3 (*p* < 0.01). [^68^Ga]Ga-PKB3 had a twofold higher (*p* < 0.0001) activity uptake in the pancreas, a GRPR-expressing organ, compared to [^68^Ga]Ga-PKB2. The tumour activity uptake was 16 ± 3%IA/g for [^68^Ga]Ga-PKB2 and 17 ± 2%IA/g for [^68^Ga]Ga-PKB3 (Fig. 4A).

[^68^Ga]Ga-PKB2 showed 1.8-fold higher tumour-to-blood ratio than [^68^Ga]Ga-PKB3. All other organs showed similar tumour-to-organ ratios for both [^68^Ga]Ga-PKB2 and [^68^Ga]Ga-PKB3. The highest T/O for both radiopeptides was observed in muscles. [^68^Ga]Ga-PKB2 and [^68^Ga]Ga-PKB3 had high tumour-to-liver and tumour-to-bone (primary metastatic sites) ratios (Fig. 4B).

**Fig. 5:**
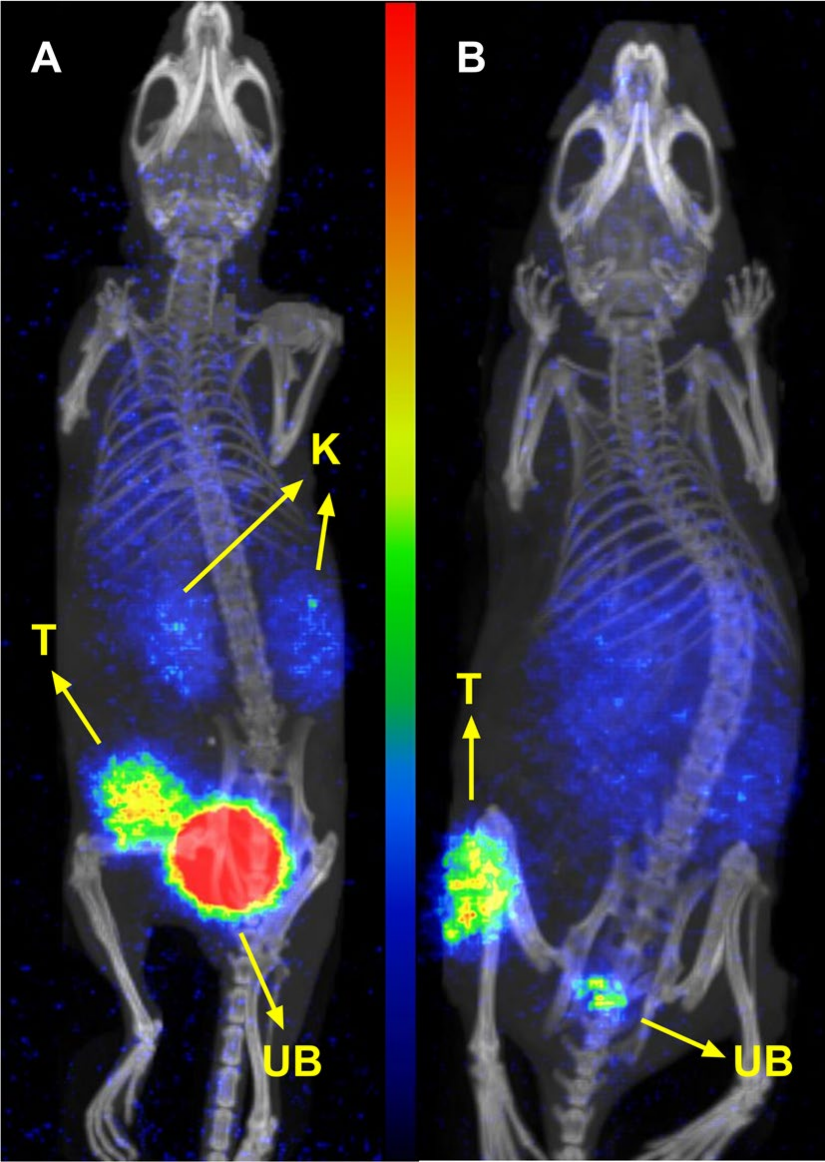
PET/CT images of [^68^Ga]Ga-PKB2 (**A**) and [^68^Ga]Ga-PKB3 (**B**) at 2 h pi in PC-3 xenografted mice. T indicates tumour, K indicates kidneys, and UB indicates urinary bladder. The scale of the colour intensity is in kBq/ml with maximum intensity at 50.8 kBq/ml

The PET/CT images for [^68^Ga]Ga-PKB2 and [^68^Ga]Ga-PKB3 were acquired at 2 h pi in mice bearing PC-3 xenografts (Fig. 5). The images of both radiopeptides were concordant with the biodistribution results, clearly visualizing the tumours.

## Discussion

The development of diagnostic radiopharmaceuticals represents a significant advancement in precision medicine, offering the potential for personalized treatment strategies. The clinical success of the radiotheranostics approach has been demonstrated with the approved radiotheranostic pairs, such as [^177^Lu]Lu-DOTA-TATE/[^68^Ga]Ga-DOTA-TATE, targeting somatostatin receptors, and [^177^Lu]Lu-PSMA-617/[^68^Ga]Ga-PSMA-11, targeting prostate-specific membrane antigen (PSMA) (Dadgar et al. 2023; Rahbar et al. 2016; Hennrich and Eder 2022, 2021). The GRPR is another well-studied target for theranostics (Reubi et al. 2002; Patel et al. 2006). Currently, there are no GRPR-targeting radiopharmaceuticals approved for clinical use, however several GRPR-targeting agents have been tested clinically for imaging and TRT (Ninatti et al. 2025; Ali 2025).

Peptides are promising vectors for targeting theranostics due to their high specificity, low production cost, rapid clearance from non-target organs, and efficient tissue penetration. However, the development of peptide-based radiopharmaceuticals also faces some challenges, including low stability in the blood circulation and high sensitivity to even minor structural modifications (Chakraborty et al. 2023). Therefore, designing a metabolically stable GRPR-targeting peptide with high affinity is essential to ensure sufficient delivery of radionuclides to the target, both for imaging sensitivity and TRT efficacy.

We previously developed the therapeutic radiopeptide [^177^Lu]Lu-AU-RM26-M2, which showed high in vivo stability with 76% intact peptide detected in blood 5 min pi (Kanellopoulos et al. 2025). We further introduced a MetTrp in position 8 of the peptide sequence to further improve in vivo stability, and reported preclinical characterisation of [^177^Lu]Lu-PKB2 (Obeid et al. 2025). The metabolic stability of the new variants was notably improved, while they maintained high affinity for GRPR. In addition, [^177^Lu]Lu-PKB2 and [^177^Lu]Lu-PKB3 showed respectively 1.7- and 1.9-fold higher tumour activity uptake at 4 h pi than [^177^Lu]Lu-AU-RM26-M2, and activity uptake was highly retained at 24 h pi, making both variants promising candidates for TRT.

In this study, we aimed to evaluate PKB2 and PKB3 labelled with the PET radionuclide Ga-68 (t_1/2_ = 68 min) to assess their suitability as diagnostic partners in a Lu-177/Ga-68 theranostic pair. Both radionuclides are trivalent metals that can form complexes with DOTAGA or DOTA chelators (Miller et al. 2022). Despite this, Ga^3+^ and Lu^3+^ differ in ionic radii and coordination geometries, which may result in different in vivo behaviour of the same compound labelled with ^68^Ga or ^177^Lu (Lymperis et al. 2018; Miller et al. 2022). This underlines the importance of characterizing both ^68^Ga- and ^177^Lu-labelled analogues to ensure a matched biological behaviour in a theranostic setting.

The peptides PKB2 and PKB3 were successfully radiolabelled with Ga-68, showing high metal-chelate stability. Upon complexation with Ga-68, both DOTAGA and DOTA chelators are expected to have a similar distorted octahedral coordination geometry. Although Ga-68 complexes with these chelators are generally less stable than those formed with the cyclic chelator NOTA (1,4,7-triazacyclononane-1,4,7-triacetic acid), the short physical half-life of Ga-68 (T_1/2_ = 68 min) and the short biological half-life of the tested peptides (activity concentration in blood was below 0.25%IA/g at 2 h pi for peptides labelled with Lu-177 (Obeid et al. 2025)) usually preclude the negative effect of in vivo instability of the metal chelate (Wadas et al. 2010). This is an essential characteristic for a diagnostic radiopeptide, as unbound Ga-68 tends to accumulate in bone tissue, a frequent metastatic site of GRPR-positive malignancies (e.g. prostate and breast cancers) (Gandaglia et al. 2014; Patanaphan et al. 1988). In this study, the high radiolabelling stability and absence of elevated bone uptake in vivo concordantly support the sufficient in vivo robustness of the ^68^Ga-DOTA/DOTAGA complexes.

[^68^Ga]Ga-PKB2 and [^68^Ga]Ga-PKB3 were compared in vitro with AU-RM26-M2 labelled with Ga-68 because it shares the same peptide sequence as PKB2 and PKB3, differing only by the absence of the MetTrp^8^ modification. Moreover, AU-RM26-M2 has demonstrated higher metabolic stability relative to its DOTA analogue (Obeid et al. 2025; Kanellopoulos et al. 2025). [^68^Ga]Ga-PKB2, [^68^Ga]Ga-PKB3, and [^68^Ga]Ga-AU-RM26-M2 demonstrated a slow GRPR-mediated internalization in PC-3 cells, indicating a typical GRPR radioantagonist profile (Cescato et al. 2008). Notably, the total activity uptake of the DOTA-conjugated radiopeptide [^68^Ga]Ga-PKB3 was significantly (*p* < 0.0001) higher than both DOTAGA-carrying radioconjugates. This behaviour was in agreement with the results of the competitive binding assay, where ^nat^Ga-PKB3 demonstrated a better GRPR-affinity than ^nat^Ga-AU-RM26-M2 and ^nat^Ga-PKB2. Similar trends were observed for the ^177^Lu-labelled analogues, with [^177^Lu]Lu-PKB3 having better K_D_ values than the DOTAGA-conjugated radiopeptides (Obeid et al. 2025; Kanellopoulos et al. 2025). These data are in agreement with previous reports, which indicate that positive charges at the N-termini of GRPR-targeting analogues enhance affinity towards the receptor, while negative charges reduce it (Abouzayed et al. 2021). Therefore, the better GRPR binding affinity of PKB3 labelled with trivalent metals can be attributed to the neutral charge at the N-terminus resulting from the substitution of the DOTAGA chelator with DOTA (Maina et al. 2017).

The better GRPR affinity for the DOTA-containing peptide is consistent with the biodistribution results, where [^68^Ga]Ga-PKB3 demonstrated a 1.5-fold higher activity uptake in the pancreas (GRPR-expressing organ) than [^68^Ga]Ga-PKB2. In tumours, [^68^Ga]Ga-PKB3 and [^68^Ga]Ga-PKB2 had similar activity uptake. This may be explained by the biological differences between tissues: the pancreas has relatively high GRPR expression and good vascularization, while PC-3 tumours often have spatially heterogeneous GRPR expression and a reduced blood perfusion (Sano et al. 2004; Muratore et al. 2021; Ma and Waxman 2009; Cornelio et al. 2007). In the organs with low expression of GRPR, like the stomach (Xiao et al. 2001) and small intestines (Monstein 2006), activity uptake of [^68^Ga]Ga-PKB2 and [^68^Ga]Ga-PKB3 was low. [^68^Ga]Ga-PKB3 showed a 1.8-fold lower renal activity uptake than [^68^Ga]Ga-PKB2, resulting in a more favourable tumour-to-kidney ratio. The difference in renal uptake could be explained by the charge difference between the [^68^Ga]Ga-DOTAGA (− 1 charge) and [^68^Ga]Ga-DOTA (neutral charge) complexes, as charges at the N-terminus are known to increase renal uptake (Roode et al. 2024). Conversely, negatively charged N-termini can reduce unspecific hepatic uptake, which may explain the tendency of [^68^Ga]Ga-PKB2 to show a lower liver accumulation than [^68^Ga]Ga-PKB3 (Rinne et al. 2019). Both radiopeptides had fast clearance, showing high tumour-to-blood, -liver, -muscle, and -bone ratios.

The biodistribution profiles of [^68^Ga]Ga-PKB2 and [^68^Ga]Ga-PKB3 were generally similar to those of their ^177^Lu-labelled counterparts (Table S1) (Obeid et al. 2025). The ^68^Ga-labelled peptides consistently demonstrated higher activity uptake in non-target organs compared to the ^177^Lu-labelled analogues, such as the spleen, lungs, and liver, resulting in a higher background signal, This trend was observed for the theranostic pairs [^68^Ga]Ga-/[^177^Lu]Lu-SB3 and [^68^Ga]Ga-/[^177^Lu]Lu-NeoBOMB1, where the ^68^Ga-labelled analogues demonstrated a higher off-target accumulation compared to their therapeutic counterparts (Lymperis et al. 2018; Dalm et al. 2017). Such differences, as mentioned previously, may be explained by differences in the physicochemical properties of the radiometal complexes (Miller et al. 2022). The higher off-target uptake and background signal on Ga-68 PET could potentially reduce lesion detectability or complicate dosimetry-based treatment planning. However, clinical experience with [^68^Ga]Ga-DOTA-TATE and [^177^Lu]Lu-DOTA-TATE suggests that, despite such biodistribution differences, Ga-68 imaging generally provides adequate information for patient selection for TRT (Stenvall et al. 2022).

Comparisons with preclinical data from clinically tested [^68^Ga]Ga-labelled GRPR-targeting peptides, including [^68^Ga]Ga-NeoBOMB1 (Dalm et al. 2017), [^68^Ga]Ga-RM2 (Mansi et al. 2011), and [^68^Ga]Ga-NOTA-PEG_3_-RM26 (Varasteh et al. 2014) would be of considerable interest. Such comparisons must be interpreted with caution, as the peptides were tested using different mouse strains, PC-3 cell batches, and varying injected activities and peptide masses. At 2 h pi, the pancreas activity uptake was lower for [^68^Ga]Ga-PKB2 (4.4%IA/g), [^68^Ga]Ga-PKB3 (8.7%IA/g) and [^68^Ga]Ga-NOTA-PEG_3_-RM26 (3.9%IA/g) than for [^68^Ga]Ga-NeoBOMB1 (22.7%IA/g) and [^68^Ga]Ga-RM2 (16%IA/g), both of which had tumour-to-pancreas ratios lower than 1 (Dalm et al. 2017; Mansi et al. 2011; Varasteh et al. 2014). Liver activity uptake for [^68^Ga]Ga-PKB2 and [^68^Ga]Ga-PKB3 was respectively 3.5-fold and twofold lower than that of [^68^Ga]Ga-NeoBOMB1 (Dalm et al. 2017). Of note, [^68^Ga]Ga-RM2 had the lowest overall liver activity uptake, an essential property for detecting liver metastases, which is a common metastatic organ for many cancers (e.g. breast cancer) (Mansi et al. 2011; Horn et al. 2020). Kidney activity uptake for [^68^Ga]Ga-PKB3 was comparable to [^68^Ga]Ga-NeoBOMB1, and 2.6-fold higher than the renal activity uptake of [^68^Ga]Ga-NOTA-PEG_3_-RM26 and twice that of [^68^Ga]Ga-RM2 (Dalm et al. 2017; Mansi et al. 2011; Varasteh et al. 2014). The diagnostic potential of [^68^Ga]Ga-PKB2 and [^68^Ga]Ga-PKB3 was confirmed with the PET/CT images of PC-3 bearing xenografts, where the tumours were clearly visualized. Therefore, based on these comparisons, [^68^Ga]Ga-PKB2 and [^68^Ga]Ga-PKB3 are expected to have promising clinical performances.

## Conclusions

PKB2 and PKB3 were successfully labelled with Ga-68, and their biodistribution profiles were in good agreement with their ^177^Lu-labelled counterparts. The PET/CT images confirmed their potential as PET tracers. These findings highlight that [^68^Ga]Ga-PKB2 and [^68^Ga]Ga-PKB3 are promising diagnostic counterparts to their ^177^Lu-labelled analogues, supporting their theranostic application as a Lu-177/Ga-68 pair.

## Supplementary Information

Supplementary Material 1 (DOCX 30 KB)

## Data Availability

All data generated of analysed during this study are present in the current manuscript and the accompanying supplementary data.

## Abbreviations

AU-RM26-M2: DOTAGA-PEG2-Pip-D-Phe-Gln-Trp-Ala-Val-Sar-His-Sta-Leu-NH_2_
PEG_2_: 8-Amino-3,6 dioxa-octanoic acid
Pip: 4-Amino-1-carboxymethyl-piperidine
Sar: Sarcosine
DOTAGA: (1,4,7,10-tetra(carboxymethyl)-1,4,7,10-tetraazacyclo-dodecane-1-glutaric acid
RM26: D-Phe-Gln-Trp-Ala-Val-Gly-His-Sta-Leu-NH_2_
PKB2: DOTAGA-PEG2-Pip-D-Phe-Gln-MetTrp-Ala-Val-Sar-His-Sta-Leu-NH_2_
MetTrp: α-Methyl L Tryptophan
PKB3: DOTA-PEG2-Pip-D-Phe-Gln-MetTrp-Ala-Val-Sar-His-Sta-Leu-NH_2_
DOTA: 1,4,7,10-Tetraazacyclododecane-1,4,7,10-tetraacetic acid
%IA: Percentage of injected activity
%IA/g: Percentage of injected activity per gram of tissue
TRT: Targeted radionuclide therapy
SPECT: Single photon emission computed tomography
PET: Positron emission tomography
CT: Computed tomography
BBN: Bombesin
GRPR: Gastrin releasing peptide receptor
NEP: Neutral endopeptidase or neprilysin
SB3: DOTA-p-aminomethylaniline-diglycolic acid-d-Phe-Gln-Trp-Ala-Val-Gly-His-Leu-NHEt
DB15: N_4_-AMA-DGA-[DPhe^6^,Sar^11^,LeuNHEt^13^]BBN(6–13); N_4_: 6-carboxy-1,4,8,11-tetraazaundecane, AMA: aminomethyl-aniline, DGA: diglycolic acid
AMTG: DOTA-Pip-D-Phe-Gln-MetTrp-Ala-Val-Gly-His-Sta-Leu-NH_2_
iTLC: Instant thin layer chromatography
RCP: Radiochemical purity
HPLC: High performance liquid chromatography
T/O: Tumor to organ ratio
