# Supplementary material for: Exploring the theranostic potential of two metabolically stable GRPR-targeting peptides labelled with Ga-68 for PET imaging
Source: EJNMMI Radiopharm Chem. 2026 Feb 17;11:13. doi: 10.1186/s41181-026-00431-5 (PMC12929764; doi:10.1186/s41181-026-00431-5)
Supplement: Supplementary file 1 — Supplementary Material 1 (DOCX 30 KB) [file 41181_2026_431_MOESM1_ESM.docx]

**Exploring the theranostic potential of two metabolically stable GRPR-targeting peptides labelled with Ga-68 for PET imaging**

**Karim Obeid^1^, Ekaterina Bezverkhniaia^1^, Vladimir Tolmachev^2^, Anna Orlova^1, 3^, Panagiotis Kanellopoulos^1*^**

^1^ Department of Medicinal Chemistry, Uppsala University, 751 83 Uppsala, Sweden;

^2^ Department of Immunology, Genetics and Pathology, Uppsala University, 751 83 Uppsala, Sweden;

^3^ Science for Life Laboratory, Uppsala University, 752 37 Uppsala, Sweden

* Corresponding author

Supplementary Material

**Table S1.** Comparison of the biodistribution profile of PKB2 and PKB3 labelled with Ga-68 vs Lu-177 at 2 h pi in PC-3 xenografted mice. Data are presented as mean ± SD (n = 4) of the individual %IA/g values; exceptions are GI and Carcass, which are given in %IA.

| **Tissue (%IA/g)** | **[^177^Lu]Lu-PKB2*** | **[^68^Ga]Ga-PKB2** | **[^177^Lu]Lu-PKB3*** | **[^68^Ga]Ga-PKB3** |
| --- | --- | --- | --- | --- |
| **Blood** | 0.13 ± 0.05 | 0.24 ± 0.03 | 0.22 ± 0.04 | 0.51 ± 0.07 |
| **Lungs** | 0.25 ± 0.08 | 3.8 ± 0.6 | 0.6 ± 0.3 | 3.6 ± 1.3 |
| **Liver** | 0.24 ± 0.05 | 2.4 ± 0.3 | 0.47 ± 0.05 | 3.7 ± 0.6 |
| **Spleen** | 0.15 ± 0.07 | 1.6 ± 0.3 | 0.4 ± 0.2 | 2.6 ± 0.7 |
| **Pancreas** | 7.3 ± 3.0 | 4.4 ± 1.9 | 11.9 ± 2.1 | 8.7 ± 1.8 |
| **Stomach** | 1.8 ± 0.5 | 1.8 ± 0.6 | 2.5 ± 0.2 | 2.9 ± 1.4 |
| **Small Int.** | 0.8 ± 0.5 | 1.0 ± 0.5 | 2.2 ± 0.6 | 2.1 ± 1.3 |
| **Kidneys** | 8.9 ± 2.5 | 7.7 ± 1.7 | 7.4 ± 1.0 | 4.5 ± 0.4 |
| **Tumor** | 15.8 ± 3.7 | 15.7 ± 2.7 | 23.8 ± 2.5 | 17.2 ± 1.8 |
| **Muscle** | 0.07 ± 0.06 | 0.08 ± 0.02 | 0.09 ± 0.02 | 0.09 ± 0.01 |
| **Bone** | 0.13 ± 0.07 | 0.25 ± 0.07 | 0.16 ± 0.03 | 0.3 ± 0.1 |
| **GI** | 1.5 ± 0.4 | 1.3 ± 0.3 | 2.0 ± 0.2 | 2.4 ± 0.6 |
| **Carcass** | 1.8 ± 1.3 | 2.7 ± 0.6 | 2.7 ± 0.8 | 3.1 ± 0.1 |
| * Values are taken from previously published data (26) | | | | |
